# Supplementary material for: Lateral Cephalometric Analytical Uses for Temporomandibular Joint Disorders: The Importance of Cervical Posture and Hyoid Position
Source: Int J Environ Res Public Health. 2022 Sep 4;19(17):11077. doi: 10.3390/ijerph191711077 (PMC9518302; doi:10.3390/ijerph191711077)
Supplement: Supplementary file 1 [file ijerph-19-11077-s001.zip › ijerph-1872883-supplementary.pdf]

Table S1. Risk of bias summary for non-RCTs Non-randomized Study of Intervention (NRS): review authors' judgements about each risk of bias item for each included study.

STUDY TYPE: NRS								
**Author (Year)**	**Bias Due To Confounding (Other Factors Such As Osteoarthiritis, Rheumatoid Arthritis, History Of Injury Of The Jaw, History Of Bruxism, Connective Tissue Disease, Previous Orthodontic Treatment)**	**Bias In Selection Of Participants Into Study**	**Bias In Classification Of Interventions**	**Bias Due To Deviations From Intended Intervention**	**Bias Due To Missing Data**	**Bias In Measurement Of Outcomes**	**Bias In Selection Of The Reported Result**	**Overall**
1. (Huggare and Raustia 1992) [14]	Critical No data regarding confounding variables.	SERIOUS No consecutive patients. No period of inclusion was stated.	LOW Treatment clearly stated.	LOW No co-interventions	Serious No follow-up period was stated.	Moderate Used Helkimo index. No masking strategy specified.	LOW Clinical and radiological assessment is well documented.	Critical
2. (Moya et al. 1994) [17]	Critical No data regarding confounding variables.	SERIOUS No consecutive patients. No period of inclusion was stated.	LOW Treatment clearly stated.	LOW No co-interventions	LOW No missing data.	Moderate No masking strategy specified.	LOW Clinical and radiological assessment is well documented.	Critical
3. (Santander et al. 2014) [21]	LOW All variables were clearly stated and investigated.	Moderate Patients included were not consecutive. It excludes male patients. No period of inclusion was stated.	LOW Treatment clearly stated.	LOW No co-interventions	LOW No missing data.	Moderate No masking strategy specified.	LOW Clinical and radiological assessment is well documented.	Moderate
4. (Kang 2020a) [18]	LOW All variables were clearly stated and investigated.	LOW No bias in selection of participants.	LOW Treatment clearly stated.	LOW No co-interventions	LOW No losses of information across the study.	Moderate No masking strategy specified.<br><br>Inter-investigator reliability was checked.	LOW Clinical and radiological assessment is well documented.	Moderate
5. (Kang 2020b) [19]	Serious Inclusion of patients with osteoarthiritis.	Low No bias in selection of participants.	LOW Treatment clearly stated.	LOW No co-interventions	Low No losses of information across the study.	Modetate No masking strategy specified. Inter-investigator	LOW Clinical and radiological assessment is well documented.	Serious

						reliability was checked.		
6. (Kim et al. 2020) [20]	LOW All variables were clearly stated and investigated.	LOW No bias in selection of participants.	LOW Treatment clearly stated.	LOW No co-interventions	LOW No losses of information across the study.	Moderate No masking strategy specified.	LOW Clinical and radiological assessment is well documented.	Moderate
